# Cellular autophagy, the compelling roles in hearing function and dysfunction

**DOI:** 10.3389/fncel.2022.966202

**Published:** 2022-09-30

**Authors:** Huanzhi Wan, Yuanyuan Zhang, Qingquan Hua

**Affiliations:** ^1^Department of Otolaryngology-Head and Neck Surgery, Renmin Hospital of Wuhan University, Wuhan, China; ^2^Research Institute of Otolaryngology-Head and Neck Surgery, Renmin Hospital of Wuhan University, Wuhan, China

**Keywords:** autophagy, auditory cells, cochlea, sensorineural hearing loss, auditory pathway

## Abstract

Sensorineural hearing loss (SNHL) is currently a major health issue. As one of the most common neurodegenerative diseases, SNHL is associated with the degradation of hair cells (HCs), spiral ganglion neurons (SGNs), the stria vascularis, supporting cells and central auditory system cells. Autophagy is a highly integrated cellular system that eliminates impaired components and replenishes energy to benefit cellular homeostasis. Etiological links between autophagy alterations and neurodegenerative diseases, such as SNHL, have been established. The hearing pathway is complex and depends on the comprehensive functions of many types of tissues and cells in auditory system. In this review, we discuss the roles of autophagy in promoting and inhibiting hearing, paying particular attention to specific cells in the auditory system, as discerned through research. Hence, our review provides enlightening ideas for the role of autophagy in hearing development and impairment.

## Introduction

As a key sensory basis for communication, hearing plays an essential role in the development of language and mental functions, and therefore, hearing loss can lead to a battery of economic difficulties and psychosocial problems (Shan et al., 2020; Yigider et al., 2020). For example, in individuals over 65 years old, hearing loss is a leading contributor to disability, which have important implications in the loneliness, social isolation, and cognitive decline (Lin et al., 2013; Shukla et al., 2020; Ge et al., 2021). Data from the WHO show that approximately one-half billion people worldwide have disabling hearing loss, and the incidence is expected to rise to 1 in 10 people by 2050 (Chadha et al., 2021). Therefore, hearing loss has come to be a major global health concern (Wilson et al., 2017; Campos and Launer, 2020).

Hearing loss can be categorized as conductive or sensorineural, which difference lies in the impairment of sound transmission or perception, and when both forms are evident, the condition is categorized as mixed (Cunningham and Tucci, 2017; Anastasiadou and Al, 2020). Caused by degenerative processes associated with aging, ototoxic drugs, noise exposure, and genetic mutations, SNHL is the most common type of hearing loss, and it is associated with a decrease in hearing sensitivity and an increase in hearing thresholds. Age-related hearing loss (AHL) is mainly caused by age-related degeneration at various auditory sites, which involves a gradually reduced hearing capacity and poor speech discernibility that is initially perceived in noisy environments. Many chemicals and clinical medications, such as aminoglycoside antibiotics and cisplatin, are known to induce deleterious ototoxic side effects. Loud sounds are ubiquitous in modern life and can damage hearing acuity, and loud noise exposure can result in temporary threshold shifts (TTSs) or permanent threshold shifts (PTSs) through direct mechanical stress and stress-induced molecular changes. Furthermore, researchers have indicated that certain genetic defects including mutations can lead to SNHL. Although SNHL is a common disease, SNHL pathogenetic mechanisms and interventions remain to be elucidated.

In the past decade, the field of autophagy research has grown exponentially (Mizushima, 2018). Since the breakthrough discovery of the molecular mechanism of autophagy by Yoshinori Ohsumi, who was awarded the Nobel Prize in 2016, considerable attention has been directed toward the role of physiopathological autophagy in various diseases (Levine and Kroemer, 2019). To maintain homeostasis, cells have evolved a self-regulating quality control system that enables adaptation to nutrient deprivation, metabolic stress, damaging challenges, and development or differentiation processes (Pohl and Dikic, 2019; Gross and Graef, 2020; Xiong et al., 2020; Yun et al., 2020). Given the prominent role of autophagy in organisms, it is important to investigate the extent to which autophagy contributes to hearing loss. Because autophagy is a programmed cell death process (Wu et al., 2020), several studies have been performed to determine whether interfering with autophagy may be potentially useful as a therapeutic strategy in SNHL. The results of these studies have increasingly shown that autophagy is connected to SNHL caused by ototoxic drugs, noise exposure, aging factors, and other causes.

## Autophagy mechanisms

**FIGURE 1:**
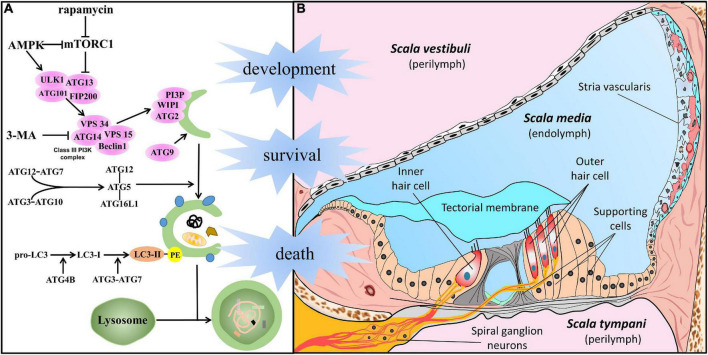
Autophagy in the cells of the auditory system. **(A)** In response to cellular stress, autophagy is promoted by AMPK or suppressed by mTORC1 and initiated by the activation of the ATG1/ULK kinase complex. After the assembly of multiprotein complexes (including Beclin1 and VPS34), the class III phosphatidylinositol 3-phosphate complex (PI3KC3) is generated to act in phagophore formation. Membrane sources from organelles and the production of PI3KC3 promote membrane elongation by recruitment of the ATG2–WIPI (ATG18 in yeast) complex. In addition, ATG9 vesicles, the transmembrane core ATG protein, contribute to the membrane supply. Underscoring the importance of the ATG complex, ATG12 conjugates with ATG5 and ATL16L1, and the LC3 subfamily conjugates with PE, resulting in an expanded phagophore engulfing autophagic cargo *via* autophagy receptors and the completion of double-membrane autophagosomes. Eventually, it gives rise to the maturation of autophagolysosomes and degradation following fusion with lysosomes. **(B)** Structure of the cochlea, especially meticulous cells in the scala media, filled with endolymph. The crucial part of auditory perception, hair cells (including IHC and OHC), are located on the basilar membrane and surrounded by epithelia-supporting cells in the Organ of Corti. Sensory HCs make synaptic connections with SGNs whose dendrites form connections with the cochlear nucleus. The stria vascularis lies on the lateral wall, and work with supporting cells plays an important role in cochlear homeostasis and acoustic function.

In general, among the types of autophagy, the three most common forms are macroautophagy, microautophagy, and chaperone-mediated autophagy. Macroautophagy, hereafter referred to as autophagy, is the major type of autophagy and is regarded as a classical degradation pathway. Degradation pathway in cells under stress is vital for preventing several clinical conditions among which cancer, neurodegeneration, and SNHL (Levy et al., 2017; Fu and Chai, 2019; Djajadikerta et al., 2020; Yang and Klionsky, 2020). Significant progress has been made in understanding the features of autophagy, and a series of genes named autophagy-related genes (ATG) have been identified in this process (Figure 1A). In response to the aforementioned cell stress, the ATG1/ULK kinase complex activates autophagy. After the assembly of multiprotein complexes (including Beclin1 and VPS34), phosphatidylinositol 3-phosphate (PI3P) is generated and is involved in phagophore formation. Organelle membrane sources and PI3P production promote membrane elongation through the recruitment of the ATG2–WIPI (ATG18 in yeast) complex. In addition, ATG9 vesicles, consisting of transmembrane core ATG protein, contribute to the expanding phagophore membrane. Underscoring the importance of the ATG complex, ATG12 is conjugated to ATG5 and ATL16L1, and LC3 subfamily proteins are bound to phosphatidylethanolamine (PE), resulting in an expanding phagophore that engulfs autophagic cargo *via* autophagy receptors and the completion of double-membrane autophagosomes. Eventually, autophagosomes fuse with lysosomes, and thus mature into autophagolysosomes, in which the autophagy cargo is degraded (Glick et al., 2010; Morishita and Mizushima, 2019; Mizushima, 2020).

To reduce cell stress, reactive oxygen species (ROS) and damaged organelles, can be removed through the autophagy mechanism described. As expected, previous reviews have suggested that in SNHL of various etiologies, autophagy leads to a certain antioxidative effect (He et al., 2018; Ye et al., 2018). However, these reviews mainly discussed different categories of hearing loss, not diverse structures or cells involved in hearing loss. Autophagy in auditory system diseases is cell-specific and difficult to generalize on the basis of type of deafness. Therefore, increasing attention has been directed to the significance of cell specificity in hearing loss (Takeda et al., 2018; McGovern et al., 2019; Roccio et al., 2020; van der Valk et al., 2021). Herein, we update the progress in understanding autophagy in auditory development and hearing loss, uniquely focusing on cellular components. Cognition of cellular state not only can lead to a better understanding of the specific role of autophagy in physiological and pathological auditory systems but can also indicate future directions in the work of this field.

## Physiology and pathology of the auditory system and autophagy participation

The cochlea, a mammalian sensor stimulated by environmental sounds, is a sophisticated and minute structure (Raphael and Altschuler, 2003; Stephenson, 2012; Iyer et al., 2016; Wright and Horn, 2016; Fettiplace, 2017). Figure 1B shows that the membranous cochlea comprises three ducts filled with endo/perilymph. The membranous cochlear duct, also called scala media, is the middle duct, and plays the most important role in sound sensing. The scala media is bounded by the vestibular membrane (on the superior side) and basilar membrane (on the basal side). The spiral ligament and stria vascularis comprise the lateral wall. Located on the basilar membrane, the spiral organ, also known as the organ of Corti, includes inner/outer hair cells (IHC/OHC), supporting cells, and the tectorial membrane. Overall, cells in the cochlea and surrounding structures are crucial for auditory perception and transmission. A full understanding of autophagy in specific cell types has been the basis for the physiological and pathological studies described in detail below.

### Autophagy in sensory cell development

Autophagy is required during the development of multiple organisms (Allen and Baehrecke, 2020). Otocysts, also named otic vesicles, are the origins of the developing sensory organs in the early embryonic inner ear. Otic vesicle ventral cells give rise to the auditory organ consisting of HCs, several types of supporting cells and neurons, which together comprise the cochlea in mammals (Magariños et al., 2014). Interestingly, an excessive number of otic cells are generated during development and are later exquisitely cleared by regulated mechanisms such as apoptosis and senescence. Developmental senescence seems to control the balance of specific cell populations in endolymphatic sac formation and modulate the otic vesicle morphology. Autophagy provides the energy for the elimination of apoptotic cells and migration of otic neuroblasts (Aburto et al., 2012; Varela-Nieto et al., 2019). In contrast, deficient autophagy leads to aberrant inner ear development (Magariños et al., 2017).

Recently, reports cited certain deafness genes (DFNA5, DFNA59, DFNA67, and connexin26) linked to autophagy and described their roles in genetic hearing loss (Hayashi et al., 2020; Koh et al., 2022). It is conceivable that autophagy is a significant process in both antenatal and postnatal cochlear cell development. A study showed that mice deficient in the Atg5 gene presented with severe congenital hearing loss and HC degeneration, revealing that autophagy is requisite for HC morphogenesis and hearing acuity stabilization (Fujimoto et al., 2017). Similarly, a newly published study described time-dependent stereocilia damage, somatic electromotility disturbances, and synaptic ribbon degeneration in mouse OHCs due to the genetic ablation of Atg7, reconfirming the role of ATG-dependent autophagy in HC preservation and hearing (Zhou et al., 2020). Another study has proven that disruptions to autophagy are involved in impaired development of cochlear ribbon synapses between IHCs and SGNs, with reduced exocytosis by IHCs in postnatal mice before hearing onset (Xiong et al., 2020). In addition, after sevoflurane exposure *in utero*, offspring mice exhibited poor hearing, as measured by the auditory brainstem response (ABR) test, with degenerated ribbon synapses despite unchanged HCs. Remarkably, impaired autophagy has been observed in cochlear explant cultures (Yuan et al., 2020). The ATG, Becn1, Atg4, Atg5, and Atg9 genes have been reported to be expressed during mouse cochlear development and their upregulated expression has been associated with concerted inner ear functional maturity. In particular, LC3B is abundant in SGNs since the first month of life, which may signify the primary association of autophagy with SGN activity (de Iriarte Rodríguez et al., 2015). In contrast to apoptosis, autophagy is gradually increased during SGN development (Hou et al., 2020).

Although no single illustration of the role of autophagy in non-sensory cell development has been described, autophagy undoubtedly has an effect on hearing. However, more evidence is needed on cochlear cell development and autophagy in pathological auditory cells.

### Autophagy in hair cell dysfunction

**FIGURE 2:**
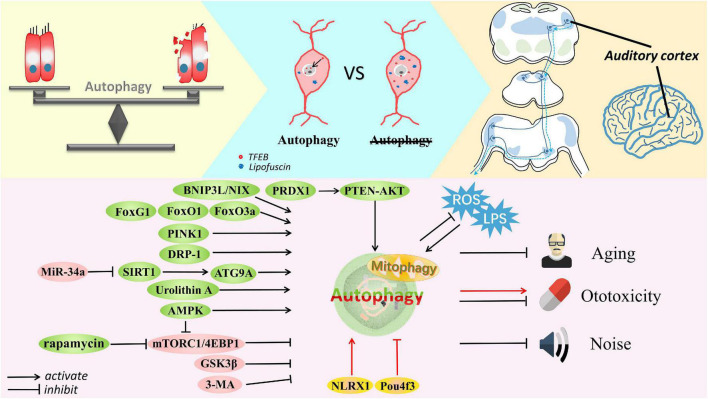
The delicate balance of autophagy in cell survival and death. Autophagy is involved in the auditory pathway, which is reviewed exactly from the peripheral to the central auditory system. Autophagy is activated by Fox family members, BNIP3L, Urolithin A, PINK1, AMPK, DRP-1, rapamycin, SIRT1, and PRDX1 but inhibited by miR-34a, 3-MA, GSK3β, and mTORC1 signaling in cells suffering from aging, noise and ototoxic drugs. Some common stresses usually exist, such as ROS and LPS. Accumulated lipofuscin and disrupted nuclear translocation of TFEB are shown in degenerated SGNs with impaired autophagy; in contrast, SGN degeneration is ameliorated after restoring autophagy and promoting TFEB nuclear translocation. Furthermore, autophagy is thought to defend against damage to the auditory cortex caused by aging or ototoxicity. Autophagy can alleviate these conditions and protect cells, although controversy remains. Activation of autophagy by Pou4f3 mutation and NLRX1 mediation can accelerate the ototoxic potential of cisplatin in hair cells and HEI-OC1 cells. Most importantly, appropriate autophagy plays a vital role in maintaining the balance of cell homeostasis.

HCs are crucial to auditory perception and they exhibit limited regenerative capacity in mammals. Specifically, IHCs specialize in the transformation of mechanical force into an electrical signal, while OHCs enhance the quality and sensitivity of the transduced signal. Damage to HCs is the major cause of SNHL. Figure 2 shows the involvement of autophagy in HC dysfunction. Some groups have found that C57BL/6 mice are susceptible to early onset hearing loss and HC loss upon upregulation of miR-34a expression; indeed, in these mice, autophagic flux is impaired (Pang et al., 2017). Specifically, ATG9A was significantly decreased after miR-34a overexpression (Yang et al., 2013) and when Sirtuin 1 (SIRT1) expression was deficient. SIRT1 is a conserved NAD-dependent deacetylase that has been confirmed as a miR-34a target (Yamakuchi et al., 2008; Imai and Guarente, 2014). A study suggested that SIRT1 deacetylates ATG9A to induce autophagy, protecting HCs and delaying AHL (Pang et al., 2019). When exposed to noise, adult CBA/J mice undergo a TTS, PTS, or sPTS (severe permanent threshold shift), with elevated lipid oxidation and protein nitration (4-hydroxynonenal and 3-nitrotyrosine, respectively, leading increased oxidative stress), mainly in OHCs. Interestingly, potentiated autophagy has been observed in OHCs exposed to moderate noise levels (such as those associated with TTSs), but autophagy was unaltered in OHCs exposed to noise associated with sPTSs, while suppressed autophagy (inhibited by 3-MA or LC3B siRNA) converted TTS to PTS and exacerbated OHC death. Additionally, activation of autophagy by rapamycin attenuated oxidative stress and thus promoted OHC survival, suggesting that autophagy modulates HC death in hearing loss and that excessive oxidative stress eliminates the benefits of autophagy (Yuan et al., 2015). Pexophagy (Germain and Kim, 2020), a selective form of autophagy, has been recently linked to pejvakin-mediated protection of HCs against noise-induced oxidative damage (Defourny et al., 2019).

As studies have progressed, increasing evidence has supported the idea that autophagy is closely associated with the alleviation of drug-induced ototoxicity of inner ear cells. Ototoxicity is an attention-attracting adverse effect of cisplatin (Yu et al., 2020), and cisplatin exposure as well as noise induce apoptosis and autophagy in HCs (Xu et al., 2018). However, the effect of autophagy on cisplatin-induced ototoxicity in HCs remains ambiguous and is debated. A study revealed an otoprotective effect of rapamycin, which reduces HC loss after cisplatin treatment by inducing autophagy (Fang and Xiao, 2014). The protective effect of autophagy induced by SIRT1 has also been described in age-dependent HC loss (Xiong et al., 2015; Pang et al., 2019). Pang et al. (2018) proposed that SIRT1-induced autophagy activation attenuates cisplatin-induced HC death in the mouse cochlea and zebrafish lateral line. Moreover, promoted autophagy in HCs accompanied by glycogen synthase kinase 3β (GSK3β) inhibition or PTEN-induced putative kinase 1 (PINK1) activation has been reported to alleviate cisplatin-induced ototoxicity. PINK1, known for its role in mitophagy, inhibits JNK pathway-related apoptosis induced in response to cisplatin injury of C57BL/6 murine cochlear explants (Yang et al., 2018a; Liu T. et al., 2019). Intriguingly, autophagy may accelerate cisplatin-induced cytotoxicity, but debate on this possibility continues (Yin et al., 2018). Recent research has indicated that mutation of the transcription factor Pou4f3 promotes apoptotic HC death by inducing autophagy in cisplatin-treated murine models of deafness (Xu et al., 2020). It is difficult to elucidate the reasons for the discrepancies in different conclusions, but they might be related to various experimental subjects. Alternatively, diversities in the timing and extent of autophagy activation may explain the differences. For example, early and moderate upregulation of autophagy has been shown to exert a protective function, but later and excessive autophagy leads to a deleterious outcome (Maiuri et al., 2007). Aminoglycoside antibiotics constitute a class of noted ototoxic medicines that includes neomycin and gentamicin, which seem to enter HCs by endocytosis through the apical membrane, basolateral membrane or mechanoelectrical transducer (MET) channel located atop stereocilia (Huth et al., 2011). A study revealed that autophagy was increased in HC explants after 2 mM neomycin treatment, and stimulation of autophagy by rapamycin rescued HCs from ROS injury and death (He et al., 2017). Other studies have shown that in gentamicin-treated cochlear HCs, multiple autophagic vacuole formed, lysosomal fusion was decreased and autophagic flux was impaired, which caused delayed onset ototoxicity (Kim et al., 2017, 2019). These studies suggest that enhancing autophagic flux might prevent aminoglycoside-induced SNHL, although we need be aware of the proapoptotic effects of autophagy overactivation.

### Autophagy in the stria vascularis and supporting non-sensory cell pathology

The stria vascularis consists of three layers of cells, including marginal, intermediate, and basal cells. It is critical for maintaining endocochlear potential (EP) and endolymph ion homeostasis and is constantly exposed to cell stress because of its high metabolic activity. It is generally assumed that the K + cycle is one of the most important mechanisms in cochlear cells; indeed, a normal stria is essential for HC mechanosensory function (Liu et al., 2016). Aberrant stria vascularis is involved in multiple types of hearing loss (Shi, 2016; Tawfik et al., 2019) and is associated with heavy oxidative burden, microvascular insults, and depletion of ion transport channels and pumps, all of which contribute to stria vascularis atrophy and, eventually, to hearing loss. Pillar, Deiters’, and Hensen’s cells are all portrayed as supporting cells with distinct morphology, location, and function, providing structural and molecular support for HCs. Furthermore, supporting cells are thought to be involved in the development and survival of HCs and SGNs and show remarkable potential for promoting HC regeneration, analogous to glia in the central nervous system (Monzack and Cunningham, 2013). Emerging evidence has suggested that the earliest signs of ototoxic drug-induced death appear in supporting cells (Ding et al., 2020); however, supporting cells are not as well characterized as sensory cells, and they deserve further investigation in efforts to prevent hearing loss.

Unfortunately, few studies have focused on autophagy related to dysfunctional non-sensory cells in hearing loss, and this aspect remains to be addressed. Poly (ADP-ribose) polymerase-1 (PARP-1) is activated under oxidative stress in marginal cells of the stria vascularis, particularly the epithelium-derived cells lining the endolymphatic surface, and subsequently induces PARP-1-dependent cell death (parthanatos). Synchronously, autophagy has been observed in these marginal cells, and autophagy suppression by 3-MA has led to aggravated parthanatos, demonstrating a prosurvival role played by autophagy in marginal cells (Jiang et al., 2018). Outer sulcus cells (OSCs) are non-sensory cells in the cochlear lateral wall, and abnormal protein aggregations in OSCs are caused by a genetic disorder with hearing loss (Pendred syndrome). It has been reported that, even at low doses, rapamycin activates autophagy in OSCs *in vivo*, thereby ameliorating anomalous protein aggregation and reducing the susceptibility of cells to disease (Saegusa et al., 2020). Additionally, as previously described (Lee et al., 2017), clusterin (CLU) has been found to be expressed in basal cells of the stria vascularis, pillar cells and Deiters’ cells in developing and mature mouse cochlea, and it has been identified as a stress-activated signaling chaperone molecule that exerts a cytoprotective effects by clearing misfolded or aggregated proteins. Studies have proven that high CLU expression in tumor cells promotes autophagy, thereby regulating the prosurvival pathway in cancer (Zhang et al., 2014; Fu et al., 2020); therefore, determination of whether CLU-related autophagy has a potential effect on the stria vascularis and supporting cells is a valid research consideration.

### Autophagy protects spiral ganglion neurons

Due to the elaborate organization of components and movements of stereocilia that protrude from the apical domain of HCs, sound impulses are transduced to the auditory nerve. There are two types of auditory nerves that arise from the differentiation of SGNs (Petitpré et al., 2018; Liu W. et al., 2019), and their peripheral processes contact HCs. Type I SGNs receive 95% afferent innervation with IHCs through ribbon synapsis development (Coate et al., 2019), and OHCs make synaptic connections with Type II SGNs. Interestingly, OHCs receive a major efferent innervation, which contributes to an efferent feedback loop with medial olivocochlear system (Guinan, 2018). With the intervention of primary sensory neurons of SGN, acoustic information is transmitted from the cochlea to the central nervous system. SGN lesions lead to SNHL due to impaired neurotrophic signaling, neurotransmitter excitotoxicity, gene deficiency and oxidative imbalances caused by aging, noise and ototoxic drugs (Nayagam et al., 2011; Liu W. et al., 2019). Herein, we explain the involvement of autophagy in SGN physiology and injury-associated hearing loss.

SGNs gradually deteriorate with HC loss. In a recently published study (Ye et al., 2019), impaired autophagic flux was found during the incipient stage of SGN degeneration. Lipofuscin and autophagic vacuoles accumulated in the cytoplasm of the degenerated SGNs in mice, and the nuclear translocation of TFEB, a transcription factor that regulates lysosomal and autophagic function, was disrupted. After intervention to promote TFEB nuclear translocation, autophagy-lysosomal function was restored, attenuating SGN degeneration. It has been demonstrated in latest reports on TFEB-mediated autophagy against aging or cisplatin-induced HC damage as well (Li et al., 2022; Xiong et al., 2022). As mentioned above, rapamycin, a specific mTORC1 inhibitor, also enhances autophagy in SGNs and ameliorates AHL in C57BL/6J mice (Liu et al., 2022). In SAMP8 mice, which are used in gerontological research, the organ of Corti and SGNs were observed to exhibit abnormalities similar to those in AHL. Specifically, a high level of LC3-II expression in SGNs was sustained from the first to the twelfth month. Notably, triggered autophagy is widely considered to play a dual role in the survival of young individuals and the death of old individuals due to cell stress (Menardo et al., 2012).

SGNs are also the targets of ototoxic drug injury. For instance, treatment with 30 μM cisplatin caused ROS activation and apoptosis of SGNs from C57BL/6 murine cochlea *in vitro* and, moreover, activation of PINK, which elicits autophagy generally. Furthermore, PINK1 silencing resulted in weakened autophagy but intensified mortality, implying that PINK1-induced autophagy might confer a protective effect in cells stimulated by cisplatin (Yang et al., 2018a). Peroxiredoxin 1 (PRDX1) is a multifunctional antioxidant and is universally expressed in the cochlea as well as in SGNs (Le et al., 2017). A recent study investigated autophagy involvement in SGN apoptosis and hearing loss induced by cisplatin and showed that PRDX1 regulated PTEN-AKT signaling to activate autophagy, thus promoting SGN survival (Liu W. et al., 2021). Another study identified prominent downregulated expression of a gene in the SGNs of rat cochlear organotypic cultures after gentamicin treatment. Ubiquitin carboxyl-terminal hydrolase isozyme L1 (Uchl1), the deficit of which leads to SGN loss and impaired autophagic flux, showed the potential to salvage autophagy in SGNs dying because of gentamicin ototoxicity (Kim et al., 2019). Furthermore, hyperactivation of the mTORC1 signaling pathway was found to be involved in the drastic gentamicin-induced reduction of SGN density and neurite outgrowth, which was restrained by rapamycin, the well-known autophagy activator (Guo et al., 2019). In addition to medicines, industrial pollution is thought to cause auditory dysfunction in humans and animals. Zhang et al. (2019) demonstrated that adult guinea pigs subjected to 60 days of chronic exposure to the heavy metal lead showed SGN loss and corresponding hearing impairment, and increased expression of ATG5, ATG6, and LC3B was found in the guinea pig brainstem after 30 days of lead exposure. Therefore, it has been suggested that autophagy possibly confers a protective effect in the early stage of lead exposure.

### Autophagy in House Ear Institute-organ of Corti 1 cells

House Ear Institute-organ of Corti 1 (HEI-OC1) is one of the rare mouse auditory cell lines. An epithelial cell line derived from a conditionally immortalized organ of Corti, the HEI-OC1 cell line expresses markers of HCs, including prestin, myosin-VIIa (MYOSIN 7a), BDNF, Atoh1, calbindin, and calmodulin (Kalinec et al., 2003). HEI-OC1 cells were originally used to investigate the potential ototoxicity or otoprotective properties of drugs in systems *in vitro* (Kalinec et al., 2016), and they are now widely used to study evaluate apoptotic pathways, inflammatory responses, gene regulation, autophagy and senescence, and oxidative and endoplasmic reticulum (ER) stress in various types of SNHL.

Autophagy correlated with AMPK has been demonstrated to regulate oxidative stress-induced premature senescence of HEI-OC1 cells, and knockdown of Atg7 has been shown to induce premature senescence by impairing autophagy (Tsuchihashi et al., 2015). Based on *in vivo* findings (Pang et al., 2017, 2019), further exploration of HEI-OC1 cells has validated that overexpression of miR-34a impairs autophagic flux by repressing ATG9A expression, whereas SIRT1 activation deacetylates ATG9A to protect against HC loss and delay AHL through autophagy recovery. As a selective autophagy subtype, mitophagy has been observed to be decreased in aged cochlea of C57BL/6 mice, which might be a result of cell damage (Oh et al., 2020; Yu et al., 2021). In other words, activation of SIRT1 or inhibition of miR-34a equally protects HEI-OC1 cells against oxidative stress and delays AHL by maintaining a balance between mitophagy and mitochondrial biogenesis (Xiong et al., 2019). Urolithin A is reported as a mitophagy activator in various mammalian cells, as well as in auditory cells. Declined mitophagy is counteracted upon Urolithin A pre-treatment in H_2_O_2_-induced senescent HEI-OC1 cells, thus maintaining mitochondrial function and preventing age-related damage (Cho et al., 2022). Dynamin-related protein-1 (DRP-1) is a GTPase that contributes to mitochondrial fission and plays a core role in mitophagy (Reddy et al., 2011; Breitzig et al., 2018). Oxidative damage induces mitochondrial dysfunction in senescent HEI-OC1 cells, while DRP-1 overexpression rescues aged cochlea from AHL by initiating mitophagy (Lin et al., 2019). Similarly, BNIP3L/NIX, a member of the BCL2 family, has been suggested to regulate mitophagy against premature senescence (Kim et al., 2021). Forkhead box (FOX) family transcription factors are influential in mammalian development and disease and are molecular sensors that regulate the interactions between the genome and signaling responses to internal or external cues (Lam et al., 2013; Tia et al., 2018; Herman et al., 2021). FoxG1 has been implicated in promoted HC survival and development in postnatal cochlea of mice (He et al., 2019). Recently, a study has demonstrated reduced autophagy levels after downregulation of FoxG1 expression in aging HEI-OC1 cells. We now have a better understanding of the role of FoxG1 in activating autophagy, which may contribute to aging cell survival (He et al., 2020, 2021). Similarly, mutation of FoxO3 (a member of a FOX family subgroup) has been reported to cause adult-onset auditory neuropathy in mice (Gilels et al., 2013). When observing external damage to cells, Liang et al. (2021) found that the FoxO3 pathway-mediated autophagy machinery possibly exerted antagonistic effects against ototoxicity. Under ER stress-induced cell damage induced by tunicamycin, a FoxO family member mediated increased autophagic levels, reducing the HEI-OC1 cell death rate (Kishino et al., 2017).

Autophagy clearly acts as a survival mechanism that protects against HEI-OC1 cell death under oxidative stress (Hayashi et al., 2015; Tsuchihashi et al., 2015); similarly, this protective effect has been effectively shown against hearing loss induced by ototoxic drugs, while impaired mitophagy aggravates cytotoxicity (Cho et al., 2021). As models used for screening the pharmacological effects of ototoxicity, studies of autophagy mechanism in HEI-OC1 cells treated with drugs have proceed apace recently (Zhao et al., 2021). It has been reported that induction of autophagy and inhibition of the p53 signaling pathway by PINK can protect HEI-OC1 cells against gentamicin-induced damage (Yang et al., 2018b), although dissention among scholars remains (Setz et al., 2018). Similar to the results with HCs, the results of *in vitro* experiments with HEI-OC1 cells have shown beneficial (Pang et al., 2018; Yang et al., 2018a; Liu T. et al., 2019) and harmful (Youn et al., 2015; Li et al., 2018; Yin et al., 2018) impacts of autophagy on cisplatin-induced ototoxicity. The latest research has indicated that autophagy-dependent ferroptosis contributes to the ototoxicity induced by cisplatin. In this study, autophagic flux was blocked by chloroquine, attenuating cisplatin injury (Jian et al., 2021). Ferroptosis is a ROS- and iron-dependent cell death. Excessive release of redox-active iron has been hypothesized to result in ferroptosis, which depends on a specific form of autophagy called ferritinophagy (Biasiotto et al., 2016; Latunde-Dada, 2017; Tang et al., 2018). After cisplatin treatment, ferritinophagy was activated and augmented iron availability, which led to ferroptotic HEI-OC1 cell death. We analyzed and discussed autophagy duality in the previous section. Remarkably, HEI-OC1 cells were used to verify the results found with in HCs *in vivo* and to explain the specific mechanism of action. Consistent with the results obtained with *in vivo* and organotypic cochlear cultures, autophagy activity, including autophagosome-lysosome fusion, was activated in HEI-OC1 cells in response to neomycin or gentamicin treatment (He et al., 2017); however, a difference was observed in other studies showing increased autophagosome formation but not lysosome fusion (Kim et al., 2017, 2019). Surprisingly, administration of the autophagic flux activator rapamycin has been universally shown to reduce ROS levels and HC death, while treatment with the autophagy inhibitor 3-MA or deletion of an autophagy gene leads to cell apoptosis.

## Autophagy in central auditory system cells

Sound information is converted from mechanical to bioelectrical signals by cochlear HCs, and then neural processing is initiated with nerve fibers consisting of SGNs. The central auditory system analyzes and deciphers sonic information, which proceeds sequentially through the cochlear nuclei, trapezoid body, superior olivary complex, lateral lemniscus, inferior colliculus, medial geniculate nucleus, and finally the auditory cortex. Due to the complexity of acoustic perception, pathological changes in the central auditory pathway, as shown in Figure 2 are worth examining. In particular, we focus on the few studies on autophagy.

Mammalian cochlear nuclei integrate acoustic and somatosensory information to localize sound sources. A study on hearing impairment in rats with diabetes reported that cochlear nucleus neurons were damaged by hyperglycemia and that the level of autophagy stimulus was increased (Xueqin et al., 2017). Another study (Fan et al., 2013) investigated the neurotoxic course of kanamycin (an aminoglycoside) in the rat dorsal cochlear nucleus, revealing aggravated injury to neurons while gradually recovered with early increases in autophagy. From the reversible damage process, we can deduce the potential protective role of promoted autophagy in kanamycin-induced neurotoxicity, but further elucidation is needed to determine the exact effect of promoted autophagy. The primary auditory cortex is the first integrative cortical area, where an elaborate network through which conscious perception and comprehension of sound and utterances are performed, and its degeneration is the major contributor to hearing loss, especially presbycusis (equally AHL). Mitophagy aids in removing ROS and maintaining mitochondrial homeostasis; however, these processes appear to be disrupted in the auditory cortex with aging. This finding indicated that mitochondrial autophagy is a promising mechanism for protecting the central auditory system against AHL (Youn et al., 2020). Using a D-galactose-induced rat model of aging, a study found that LC3 and Beclin1 were increased from young to adult in the auditory cortex because of AMPK-mTOR-ULK1 signaling, which maintained neuronal ultrastructural morphology. In contrast, these proteins were reduced at old, and the apoptosis rate and substantial neuron degeneration were increased. These results suggested antiapoptotic and antiaging functions of autophagy in the degeneration of the auditory cortex (Yuan et al., 2018). Additionally, SIRT1 has been found to play a protective role in initiating autophagy in the peripheral auditory system. A decrease in SIRT1 expression in the auditory cortex with aging has been reported, and this decrease may contribute to presbycusis (Xiong et al., 2014). Since research data have revealed that SIRT1 induces autophagy in response to survival stress, this result suggests the possibility of autophagy function in the auditory cortex. Altogether, evidence indicates that autophagy plays a role in the central auditory system, but more studies need to be performed to discern the relationship between autophagy and hearing loss in this field.

## Conclusion and outlook

Hearing loss is a major cause of disability that continues to be invisible and is therefore, a silent epidemic. As shown in Table 1, we mainly reviewed the pathological factors, genesis, and molecular mechanism of hearing loss to shed light on the effects of autophagy in specific cells of the auditory system. Notably, an increasing number of studies have been published on aural impairment; nonetheless, studies on the autophagic role have been rare to date. Mitochondrion is vital for cellular function and mitophagy has gained increasing importance in response to stress. Studies on mitophagy in SNHL are promising, note also that there is no literature reported regarding other forms of autophagy including microautophagy and chaperone-mediated autophagy, which need more exploration in auditory system. Research on hearing loss therapy is flourishing and focuses on the cell biology of the auditory system (Nishio et al., 2017; Takeda et al., 2018; Furness, 2019; Ranum et al., 2019; Hoa et al., 2020). To better understand the function of autophagy in different cell-enriched regions of the cochlea, we collated and summarized advances in autophagic knowledge from the perspective of different cells. Regardless of the disease model, research should be directed to specific cellular targets. Moreover, researchers and clinical practitioners are showing considerable interest in cellular targets of SNHL that are modulated by autophagy. Autophagy might perform diverse biological functions in different SNHL cells, even those derived from the same etiological factor. Certainly, taking a cell perspective rather than an etiological view can improve our understanding of the exact position of SNHL and development of appropriate treatments.

**TABLE 1:** Researches on the mechanism and function of autophagy in various cells of auditory system.

Cells	Mechanism (gene and molecule)	Development/diseases model	Protective/ degradative	References
HCs	ATG5 deficient	Congenital hearing loss	Protective	Fujimoto et al., 2017
	ATG7 deficient	OHC degeneration and hearing loss	Protective	Zhou et al., 2020
	Activate and inhibit autophagy, respectively by rapamycin or 3-MA	Development and maturation of cochlear ribbon synapses between IHC and SGNs	Protective	Xiong et al., 2020
	SIRT1 deacetylate ATG9A to alleviate HC loss, while MiR-34a represses it	AHL	Protective	Pang et al., 2017, 2019
	Activate and inhibit autophagy, respectively by rapamycin or 3-MA	Noise-induced hearing loss	Protective	Yuan et al., 2015
	Autophagy is activated by rapamycin	Cisplatin induced hearing loss	Protective	Fang and Xiao, 2014
	Autophagy is promoted by SIRT1 activation	Cisplatin induced hearing loss	Protective	Pang et al., 2018
	Enhanced autophagy in response to GSK3β inhibition	Cisplatin induced hearing loss	Protective	Liu T. et al., 2019
	Activation of autophagy by Pou4f3 gene mutation or knockout could induce apoptosis.	Cisplatin induced hearing loss	Degradative	Xu et al., 2020
	Autophagy is activated by rapamycin	Neomycin induced hearing loss	Protective	He et al., 2017
	Delayed onset ototoxicity caused by impaired autophagic flux	Gentamicin induced hearing loss	Protective	Kim et al., 2017, 2019
MCs	Autophagy inhibition by 3-MA exacerbates parthanatos	AHL	Protective	Jiang et al., 2018
OSCs	Autophagy is activated by rapamycin	Genetic hearing loss disorder	Protective	Saegusa et al., 2020
SGNs	Gradually upregulated autophagic activity during postnatal development	SGNs development	Protective	Hou et al., 2020
	LC3B expression is intense in adult SGNs	SGNs functional maturity	Protective	de Iriarte Rodríguez et al., 2015
	Restoring autophagic flux attenuates SGNs degeneration by promoting TFEB nuclear translocation *via* inhibiting mTOR	SGNs degeneration model	Protective	Ye et al., 2019
	Rapamycin enhanced autophagy by inhibiting mTOR activation	AHL	Protective	Liu et al., 2022
	autophagic stress and accumulated lipofuscin in SGNs of SAMP8 mice	AHL	Protective/degradative bidirectional	Menardo et al., 2012
	Autophagy is induced by PINK1	Cisplatin induced hearing loss	Protective	Yang et al., 2018a
	PRDX1 activate autophagy to attenuate cisplatin damage through activation of PTEN-AKT signaling pathways	Cisplatin induced hearing loss	Protective	Liu W. et al., 2021
	Hyperactivation of mTORC1 is restrained by rapamycin	Gentamicin induced hearing loss	Protective	Guo et al., 2019
HEI-OC1	Autophagy is activated by enhanced nuclear translocation of TFEB	Cisplatin induced hearing loss	Protective	Li et al., 2022
	Atg7 silencing resulted in premature senescence after H_2_O_2_ treatment	Premature senescence of auditory cells induced by oxidative stress	Protective	Tsuchihashi et al., 2015
	Rapamycin rescues the inhibition of TFEB nuclear translocation regulated by miR-34a/ATG9A signal, restores autophagic flux and consequently prevents cell death.	AHL	Protective	Xiong et al., 2022
	Impaired mitophagy is observed in aged cochlea	AHL	Protective	Oh et al., 2020
	Activation of SIRT1 or inhibition of miR-34a modulates autophagy	AHL	Protective	Pang et al., 2017, 2019; Xiong et al., 2019
	Mitophagy is restored upon Urolithin A pre-treatment of H_2_O_2_-induced senescent cells to exert anti-aging effects	AHL	Protective	Cho et al., 2022
	Activated DRP-1 initiate mitophagy to rescue aged cochlea	AHL	Protective	Lin et al., 2019
	BNIP3L/NIX-mediated mitophagy protects against premature senescence	AHL	Protective	Kim et al., 2021
	Activated FoxG1 expression and following autophagy upregulation helps aging cells survival	AHL	Protective	He et al., 2021
	Impaired mitophagy aggravates cytotoxicity	Cisplatin induced hearing loss	Protective	Cho et al., 2021
	FoxO3 pathway mediated autophagy acts against ototoxicity	Cisplatin induced hearing loss	Protective	Liang et al., 2021
	XBP1-FoxO1 interaction regulates autophagy within ER stress	Tunicamycin induced hearing loss	Protective	Kishino et al., 2017
	Autophagy impaired by Atg7 knockdown demolishes Keap1–Nrf2 signaling crosstalk through p62	H_2_O_2_ induced ATP depletion and oxidative stress in auditory cells	Protective	Hayashi et al., 2015
	Impaired autophagy by acetaminophen induced cell death	Acetaminophen-induced ototoxicity	Protective	Zhao et al., 2021
	Promoted autophagy and inhibited p53 by PINK1 could protect against cell damage	Gentamicin induced hearing loss	Protective	Yang et al., 2018b
	Activated autophagy in HEI-OC1 cell	Cisplatin induced hearing loss	Protective	Pang et al., 2018; Yang et al., 2018a; Liu W. et al., 2019
	Upregulation of autophagy accelerates cell death	Cisplatin induced hearing loss	Degradative	Youn et al., 2015; Li et al., 2018; Yin et al., 2018
	Ferroptosis-related autophagy contributes to cell death	Cisplatin induced hearing loss	Degradative	Jian et al., 2021
Central	Increased autophagy by hyperglycemia that damages the cochlear nucleus neurons.	Hearing loss in rats with diabetes	Degradative	Xueqin et al., 2017
	Autophagy is promoted after kanamycin treatment *via* JNK1-p-Bcl-2-Beclin-1 signaling pathway in dorsal cochlear nucleus	Impairment auditory function by kanamycin	Protective (potential)	Fan et al., 2013
	Mitophagy appears to be damaged in auditory cortex with aging	AHL	Protective	Youn et al., 2020
	LC3, BECN1, BCL-2 and BCL-xL increase at 3 months while decrease at 15 months in the auditory cortex	AHL	Protective	Yuan et al., 2018

Unsurprisingly, autophagy-related studies mainly focus on auditory sensory cells, and greater attention needs to be directed to non-sensory cells. Of these cells, autophagy in HCs has been the most widely researched in various forms of SNHL, and HEI-OC1 cells have been concurrently used for *in vitro* confirmation and supplementation of experimental results (Figure 2). Unexpectedly, we found less concrete evidence linking autophagy with hearing loss in stria vascularis and supporting cells, that is, in non-sensory cells; however, hints of a connection were found. Moreover, to fully understand hearing loss caused by aberrations in the auditory pathway (Figure 2), we briefly reviewed the function of autophagy in the central auditory system. Recognizing interrelations among unexplored systems may provide a comprehensive view of autophagy and hearing loss, and possibly other neurodegenerative diseases.

The literature has confirmed the importance of intact autophagy in the development of cells in the inner ear (Fujimoto et al., 2017; Magariños et al., 2017). In addition, autophagy may not have been given attention in studies on hearing in healthy adult mice, but under abnormal conditions caused by aging or exposure to noise or ototoxic drugs, appropriate autophagy has been shown to be particularly important in hearing conservation (Fujimoto et al., 2017; Magariños et al., 2017; Fu and Chai, 2019). For each cell type studied, including HCs, SGNs, non-sensory cells and central auditory cells, activated autophagy signaling through common or similar regulatory pathways has been shown to be beneficial under most conditions. The antioxidant effect seems to be a major role played by autophagy to protect against hearing impairment, as indicated by ROS clearance and cell survival observed in autophagy-activated cells. In addition, ameliorating organelle degradation, relieving the inflammatory response and attenuating aberrant molecular aggregation contribute to the cytoprotective effect conferred by autophagy. Given the favorable role of autophagy in most cases, many researchers agree that autophagy promotes the survival of auditory cells under stress. However, autophagy-related apoptosis remains disputed territory. Pou4f3 mutation and NLRX1-mediated autophagy or ferritinophagy can accelerate the damage to HCs treated with ototoxic drugs. Overactivation of autophagy may be associated with apoptotic effects; however, the complex relationship between autophagy and apoptosis remains unclear (Gordy and He, 2012; Doherty and Baehrecke, 2018; Yan et al., 2019). Therefore, several topics worth exploring for subsequent studies include (1) How does autophagy affect the developing auditory system? (2) Is the role of autophagy related to prosurvival or proapoptosis in pathological auditory cells? (3) Is autophagy consistent in different cells and regions of the auditory system? (4) How can we detect and modulate the dual roles of autophagy?

Overall, research on the role played by autophagy in hearing loss is in an emergent phase (He et al., 2018; Ye et al., 2018; Fu and Chai, 2019; Guo et al., 2021; Liu C. et al., 2021). Certainly, more investigation into various auditory cells, including non-sensory cells, is needed to determine the autophagic function in specific cells involved hearing loss. In summary, our review suggests cellular autophagy as a promising target and strategy for future research and clinical therapeutics in hearing loss.

## Author contributions

HW collected the data, wrote original draft, and performed data analysis. YZ performed data analysis, reviewed and edited, resourced, and supervised. QH conceived and designed, reviewed and edited, resourced, and supervised. All authors read and approved the final manuscript, contributed to the study conception and design.

## Abbreviations

ABR: auditory brainstem response
AHL: Age-related hearing loss
ATG: autophagy-related genes
CLU: clusterin
DRP-1: Dynamin-related protein-1
EP: endocochlear potential
ER: endoplasmic reticulum
GSK3 β: glycogen synthase kinase 3 β
HCs: hair cells
HEI-OC1: House Ear Institute-organ of Corti 1
IHC/OHC: inner/outer hair cells
OSCs: Outer sulcus cells
PARP-1: Poly (ADP-ribose) polymerase-1
PE: phosphatidylethanolamine
PI3P: phosphatidylinositol 3-phosphate
PINK1: PTEN-induced putative kinase 1
PRDX1: Peroxiredoxin 1
PTSs: permanent threshold shifts
ROS: reactive oxygen species
SGNs: spiral ganglion neurons
SIRT1: Sirtuin 1
SNHL: Sensorineural hearing loss
sPTS: severe permanent threshold shift
TTSs: temporary threshold shifts
Uchl1: Ubiquitin carboxyl-terminal hydrolase isozyme L1.
